# Lychee13-3634: A new lychee image dataset and classification methodological evaluation

**DOI:** 10.1371/journal.pone.0334900

**Published:** 2025-10-23

**Authors:** Shaoye Luo, Hanling Zheng, Ziyang Lin, Tingting Zeng, Miaomiao Huang, Yongyi Xiao, Antoni Grau, Jiayan Huang

**Affiliations:** 1 College of Computer and Data Science, Putian University, Putian, Fujian, China; 2 Engineering Research Center for Big Data Application in Private Health Medicine of Fujian Universities, Putian University, Putian, Fujian, China; 3 Artificial Intelligence College, Putian University, Putian, Fujian, China; 4 Faculty of Data Science, City University of Macau, Macau, China; 5 Department of Automatic Control, Robotics and Computer Vision, Polytechnic University of Catalonia, Barcelona, Spain; 6 Fujian Ruituo Information Technology Co., Ltd., Putian, Fujian, China; Kafkas University: Kafkas Universitesi, TÜRKIYE

## Abstract

The rapid and accurate classification of lychee varieties is crucial for improving production efficiency and optimizing market supply. Especially for the main production areas of lychee, efficient lychee classification is more urgent. However, there is currently no publicly available comprehensive and diverse lychee benchmark dataset for precise training of classification models. To fill this gap, this work constructs a comprehensive lychee image dataset (Lychee13-3634), which covers 13 varieties and 3634 images. Different from the general fruit datasets, which show significant differences in features between their fruit images, Lychee13-3634 highlights minor inter-class differences among various lychee varieties. Based on this dataset, we applied 20 advanced deep learning-based classification models to validate its availability and effectiveness. Meanwhile, we comprehensively evaluated and provided meaningful insights about all models. Experimental results show that EfficientNetv2 has the best classification performance with an accuracy of up to 99.90%. Besides, we further comprehensively analyzed the balance of Lychee13-3634, and the corresponding experiments demonstrate that a more balanced dataset usually leads to better classification performance of the model. In summary, Lychee13-3634 provides benchmark training data for the lychee image classification task and demonstrates the effective application of existing deep learning classification models, providing reference and inspiration for other agricultural product image recognition research. Our Lychee13-3634 and all evaluation models are available at https://github.com/jyanhuang/Lychee13-3634.

## Introduction

As one of the most popular fruits in tropical and subtropical regions, Lychee is not only loved by consumers for its sweet taste, but also attracts attention for its rich nutritional value. Precise lychee classification can accurately identify the appearance, color, and shape, effectively avoiding the subjectivity and errors associated with traditional methods, while high speed and efficiency enable faster quality inspection in production. Therefore, high accuracy and efficiency play an indispensable role in production. Recently, with the rapid development of artificial intelligence (AI) technologies, such as machine learning and deep learning [1,2] have been widely applied in image classification and identification tasks to improve the accuracy and efficiency [3–5]. For the task of lychee image classification, advanced technology can be used to achieve more accurate and faster classification, laying the foundation for the intelligent development of the lychee industry [6]. However, despite significant progress in image recognition technology [7–11] for classifying other agricultural products, research on lychee image classification still faces many challenges. Compared to other fruits, lychee has many varieties and subtle differences in appearance characteristics between different varieties, such as color, texture, shape, and so on. Traditional classification methods often rely on human experience, which is not only inefficient but also susceptible to subjective factors. Therefore, building a dataset that comprehensively covers different lychee varieties and appearance characteristics is crucial to promoting the development of lychee image classification technology.

The existing dataset containing lychee images usually only treats lychee as a rough category among many fruit classifications, which is difficult to meet the multi-variety lychee classification tasks in the market. In other words, currently, lychee datasets have the problems of limited data and incomplete variety coverage [12,13], which limits the generalization ability of machine learning and deep learning models in lychee variety recognition [14]. Besides, data imbalance also affects the recognition accuracy of the model for a few categories of lychee varieties [15]. To make up for these problems, this study innovatively proposes a comprehensive lychee dataset (Lychee13-3634) containing 13 typical varieties and 3634 images. Compared to other datasets, Lychee13-3634 is a more comprehensive and unified lychee dataset. For example, Fruits-360 has not explicitly disclosed their image resolution, while Lychee13-3634 uniformly uses a high-definition resolution of 400 × 400 pixels. This is more in line with the input requirements and helps improve the training performance of classification models. Moreover, in terms of sample balance, there is a significant difference in the number of samples for different fruit categories in Fruits-360, while the Lychee13-3634 has achieved a class imbalance rate (*IR*) of only 1.6 through careful construction, demonstrating a relatively more balanced class distribution. This is crucial for training a comprehensive and accurate lychee classification or recognition model. To sum up, through the detailed data acquisition and screening process, we ensured the accuracy and diversity of the Lychee13-3634, and provided a new benchmark and test platform for lychee image classification research.

In addition, to verify the effectiveness and reliability of the Lychee13-3634, we used 21 state-of-the-art classification models, including 6 typical ResNet-series, 10 deep learning-series, and 5 YOLO-series models for lychee image classification training. During the training, we used a series of performance indicators to comprehensively evaluate the classification performance and efficiency of each model. The results of these indicators directly reflect the performance of each model in lychee image classification. Through in-depth analysis and interpretation of these indicators, we not only understand the advantages and disadvantages of each model in the classification task, but also deeply explore the reasons for the performance differences between different models. This detailed analysis not only helps us better understand the characteristics of each model, but also provides a useful reference for future research, which helps to further promote the development of lychee image classification technology.

## Related exisiting fruit datasets

In this section, we conducted research and summarized existing commonly used fruit image datasets (including/excluding lychee images). Table 1 provides detailed information about each dataset, including publication year, classes, class number, total image number, image size, format, method type (classification, segmentation, or recognition), and including/excluding lychee images.

**Table 1:** The detailed information of different existing fruit datasets.

Datasets	year	classes	class number	image number	image size	format	method type	i/e lychee (number)
*Fruits*-360	2017	fruit & vegetable	141	94,110	100×100	JPEG	classification	Y (490)
*Fruits*-262	2021	fruit	262	225,640	non-uniform	JPEG	classification	Y (1008)
*FruitNet*	2021	fruit	6	14,700	non-uniform	JPEG	classificaiton	N
*Fruit*-*Images*	2023	fruit	9	360	non-uniform	PNG	classification	N
*Fruit*-*Classification*	/	fruit & vegetable	33	22,495	100×100	JPEG	classification	N
*Comprehensive FruitImage*	/	fruit	20	3,400	non-uniform	JPEG/ PNG	classification	Y (190)
*Lychee*13-3634 (*ours*)	2024	lychee	13	3,634	400×400	JPEG	classification/ recognition	Y (3634)

The download links for each dataset are as follows, Fruits-360 https://www.kaggle.com/datasets/moltean/fruits Fruits-262 https://www.kaggle.com/datasets/aelchimminut/fruits262 FruitNet https://www.kaggle.com/datasets/shashwatwork/fruitnet-indian-fruits-dataset-with-quality Fruits-Images https://www.kaggle.com/datasets/shreyapmaher/fruits-dataset-images Fruit-Classification https://www.kaggle.com/datasets/sshikamaru/fruit-recognition Comprehensive Fruit Image https://www.kaggle.com/datasets/evilspirit05/comprehensive-fruit-image-dataset

*Fruits-360 dataset* [12] is a public baseline dataset for classification tasks. It contains 94,110 images of 141 different fruit, vegetable, and nut classes, with 70,491 for training and 23,619 for testing, respectively. All images were taken from different angles and lighting conditions, providing a rich visual diversity. The Fruits-360 has a pure white image background, and each image contains only one complete fruit, and only 490 lychee images without further precise category labels. This data collection method guarantees the representation of color, texture, and shape, which is well suited for training and evaluating fruit classification models. Its size and diversity support the effective performance of deep learning models in fruit recognition tasks, commonly used for benchmarking and research development.

*Fruits-262 dataset* [13] is an image dataset containing 262 different categories of fruits. It is designed for computer vision tasks such as image classification and object recognition. The dataset consists of natural background images, each containing multiple fruits, with an average number of images of 861, a median of 1007, and a standard deviation of 276. Building the dataset involved crawling images from the Internet, automatic and manual filtering, and image resizing. This dataset can be used to train a convolutional neural network (CNN) model to recognize and classify different fruits. It can also serve as a valuable resource for studying computer vision and machine learning algorithms.

*FruitNet: Indian-Fruits-Dataset-With-Quality dataset* [16] address the need for high-quality fruit images. It contains 14,700 high-resolution images of 6 popular Indian fruits. The dataset is divided into 3 subfolders based on fruit quality: 1) good quality, 2) poor quality, and 3) uneven quality. Each subfolder contains images of 6 fruits: apple, banana, guava, lime, orange, and pomegranate. All images are taken with a high-end resolution camera under various backgrounds and lighting conditions and are valuable for training, testing, and validating fruit classification or reconstruction models.

*Fruits-Images dataset* [17] is widely used in computer vision and deep learning research, including 360 high-quality images of a category of common fruits, such as apples, bananas, oranges, kiwifruit, and strawberries, with a single image containing multiple fruits of the same variety. Despite the relatively small number of images, different shooting conditions and angles provide valuable training data for developing and evaluating classification models. Fruits-Images has accurate class labels, supports supervised learning tasks, and contributes to the progress of image classification techniques and the improvement of fruit recognition techniques.

*Fruit-Classifiation dataset* [18] is designed for image classification and contains 22,495 images from 33 categories such as watermelon, dragon fruit, and corn. Most of the images are with a size of 100 × 100, and there are references to Fruits-360 images that often include rotations to aid training and improve usability. The main application of this dataset is to improve the accuracy of fruit image recognition. In practice, it is common to perform preprocessing steps such as normalization and augmentation to optimize model performance.

*Comprehensive-Fruit-Image dataset* [19] is a valuable resource that provides support for image classification and recognition models. It contains 20 different fruit categories and uses specific fruit names as search criteria to carefully pick and download images from the DuckDuckGo search engine to ensure a diverse and high-quality dataset. In particular, each fruit category contains a large number of pulp images for efficient model training and testing. The images in the dataset are stored in high-resolution format and organized by fruit type for use during model training and evaluation. This dataset can be used for a variety of machine learning tasks, such as image classification and recognition, as well as enhancement of computer vision models. It is also a practical resource for students and educators in the field of machine learning and computer vision. By providing a diverse and curated collection of fruit images, this dataset aims to facilitate significant progress in the field of computer vision and machine learning. Researchers and developers can use this dataset to build robust, accurate, and efficient image recognition systems.

To sum up, the existing fruit and vegetable datasets contain few specific lychee varieties, or the variety is not detailed enough. It just exists as a fruit and vegetable category. For example, the Fruits-360 dataset, which is widely used for fruit classification, contains a large number of fruit images with distinct visual features, while lychee varieties exhibit high similarity and low inter-class differences. In contrast, lychee varieties often exhibit subtle differences in color, texture, and shape, posing unique challenges for lychee classification. This highlights the need for a specialized dataset for lychee classification. Besides, with the advancement of agricultural intelligence, fast and accurate classification of lychee varieties is of great significance for optimizing market supply, reducing labor consumption, and improving production efficiency. Effective recognition of different varieties of lychee images can meet the practical needs of agricultural production, quality control, market classification, and consumer choice. Therefore, a more refined lychee dataset needs to be proposed.

## The proposed dataset: Lychee13-3634

**Fig 1:**
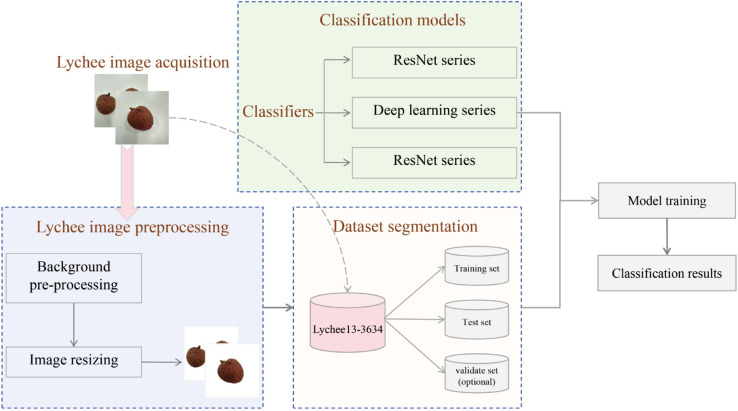
The overall flowchart for Lychee13-3634 construction and classification application. It illustrates the complete process of this work, which mainly includes four stages, i.e., lychee image acquisition, preprocessing, training-test set segmentation, and classification model training.

In this section, we first introduce lychee image acquisition and then introduce the lychee image background preprocessing. Finally, we present the overview of Lychees13-3634 and analyze the balance of it. The overall flowchart of this work is shown as Fig 1.

### Lychee image acquisition

To solve the problem of lychee non-fine-grained classification, we construct a new lychee image dataset (Lychee13-3634), which aims to provide high-quality training and testing data for the deep learning model of lychee image classification or recognition. Notably, the construction of Lychee13-3634 is mainly based on the three aspects of market prevalence, sales volume, and availability. Specifically, we selected 13 varieties widely distributed in southern China to ensure the universality and sufficient data samples for collection. Moreover, their extensive market liquidity makes sample collection convenient. Meanwhile, the existing relative researches provide reference for data annotation, analysis, and model training, which greatly improves data availability and helps to build a high-quality lychee dataset.

All the lychee images are freshly picked and taken with a high-resolution camera under indoor lighting conditions. Specifically, the capture camera is a 48-million pixel main camera with a 24 mm focal length and f/1.78 aperture. The advanced camera configurations ensure the high quality of image acquisition and provide a solid foundation for the reliability of experimental results. During shooting, special attention was paid to factors such as illumination uniformity and background conciseness to ensure clear image quality, true color, and to reflect the real appearance characteristics of lychee.

The proposed Lychee13-3634 dataset was collected by the authors themselves. All lychee image data were purchased from publicly available markets in strict compliance with relevant laws, regulations, and ethical guidelines. The purpose of the Lychee13-3634 collection is limited to scientific research, aiming to improve the accuracy and efficiency of lychee image classification and recognition.

### Lychee image preprocessing

To facilitate the training of the deep learning model, we pre-process the lychee images. Including background preprocessing, uniformizing the image size to a resolution suitable for model input (400×400 pixels). In addition, we also randomly divided the Lychee13-3634 into a training set (80%) and a test set (20%) to meet the needs of different research stages. In particular, for the specific background pre-process, we provide its pseudocode implementation in Algorithm 1.

**Algorithm 1 Background preprocessing of lychee image.**

**Require:** original lychee image *I* with size of H×W, color difference threshold *K*.

**Ensure:** processed lychee image *J*.

1: initialize a two-dimensional array *I*_*map*_ with a size of W×H

and set all elements to 2 (representing foreground), while

set the upper left corner element (*I_map_[0][0]*) to 0

(representing background).

2: *J* = *I*

3: **for** x←0 to (W−1) **do**

4:   **for** y←0 to *H*−1 **do**

5:    $C_{current}\leftarrow I[x][y]$

6:    **if** *x*<*W*−1 **then**

7:     $C_{neighbor}\leftarrow I[x+1][y+1]$

8:     D← EuclideanDistance($C_{current},C_{neighbor}$)

9:     **if** *D*<*K* and $I_{map}[x+1][y]$ == 2 and $I_{map}[x][y]==0$ **then**

10:      *J*[*x* + 1][*y*] = 255

11:     **end if**

12:    **end if**

13:    **if** *y*<*H*−1 **then**

14:     $C_{neighbor}\leftarrow I[x][y+1]$

15:     D← EuclideanDistance($C_{current},C_{neighbor}$)

16:     **if** *D*<*K* and $I_{map}[x][y+1]==2$ and $I_{map}[x][y]==0$ **then**

17:      *J*[*x*][*y* + 1] = 255

18:     **end if**

19:    **end if**

20:   **end for**

21: **end for**

22: **return** *J*

**function** EuclideanDistance(*X*, *Y*) = **return** $\sqrt{(X_{R}-Y_{R}{)}^{2}+(X_{G}-Y_{G}{)}^{2}+(X_{B}-Y_{B}{)}^{2}}$

**Noted:** This background preprocessing approach assumes uniform backgrounds and that alternative methods may be needed for complex field conditions, such as varying lighting or occlusions.

**Fig 2:**
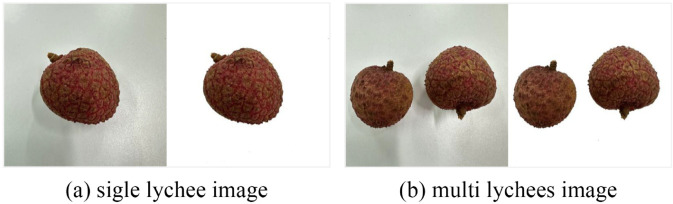
Comparison of lychee samples before and after background preprocessing. It can be seen that before processing, there are interferences such as referencing, uneven lighting, and blurring in the image background. While after processing, it illustrates clearly and uniformly, making it easy for fine-tuning in other research.

As shown in Fig 2, it provides a comparison of lychee samples before and after background preprocessing. Obviously, after background preprocessing, the noise issues such as shadows caused by unstable lighting during image capture have been balanced. This can make the dataset more standardized and unified, thereby reducing bias caused by the dataset during model training.

### Lychee13-3634 overview

**Fig 3:**
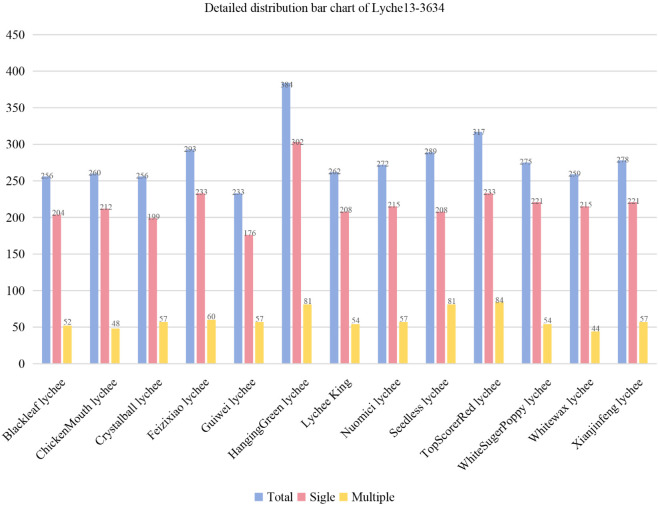
The distribution bar chart of the proposed Lychee13-3634. Each category includes different numbers of images with a single lychee and images containing multiple lychees of the same variety, and the average number of single lychee images is around 220, while the average number of multiple lychee images is around 60.

**Fig 4:**
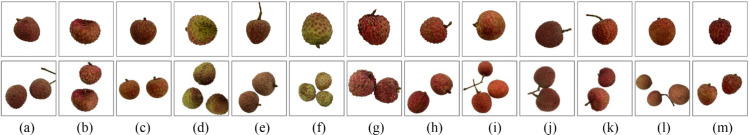
Examples of 13 different categories of single-lychee and multiple-lychees images from Lychee13-3634. (a) Blackleaf. (b) Chicken Mouth. (c) Crystalball. (d) Feizixiao. (e) Guiwei. (f) HangingGreen. (g) LycheeKing. (h) Nuomiei. (i) Seedless. (j) TopScoreRed. (k) WhiteSuggerPoppy. (l) Whitewax. (m) Xianjinfeng. The first row displays images of a single lychee of each category, while the second row displays images of multiple-lychees of the same variety. It can be seen that the differences between most categories are small and difficult to distinguish directly.

Lychee13-3634 is a comprehensive and standardized lychee image dataset, which contains 3634 images, covering 13 representative varieties. These varieties have high popularity and economic value in the market, covering the main types and variations of lychee. Table 2 and Fig 3 provide a detailed summary of Lychee13-3634. And Fig 4 shows 13 different varieties of single and multiple lychee samples, respectively.

**Table 2:** The specific details of our proposed Lychee13-3634 Dataset.

Dataset	Lychee13-3634
*Lychee Varieties*	Blackleaf_lychee, ChickenMouth_lychee,
	Crystalball_lychee, Feizixiao_lychee,
	Guiwei_lychee, HangingGreen_lychee,
	Lychee_King, Nuomici_lychee,
	Seedless_lychee, TopScorerRed_lychee,
	WhiteSugerPoppy_lychee, Whitewax_lychee,
	Xianjinfeng_lychee
*Data Format*	JPG
*Image Size*	400 × 400
*Total Number*	3634
*Application*	Image classification/recoginition/detection

To the best of our knowledge, the proposed Lychee13-3634 dataset is the first benchmark dataset for fine-grained classification of lychee to date. However, there are still certain limitations. On one hand, it does not include information on the maturity of the lychees, which may affect the model’s generalizability. On the other hand, the sizes and the production area of different lychee varieties were not recorded during data collection. This lack of information may limit the dataset’s applicability in certain research scenarios. Additionally, while efforts were made to ensure data quality, potential biases may still exist due to factors such as uneven sample distribution or variations in shooting conditions.

### Lychee13-3634 balance analysis

For further analysis, we use the imbalance rate (*IR*) [20], which is an important indicator to measure the imbalance degree of the dataset. *IR* is defined as the ratio of the number of samples in the majority class to that in the minority class. A higher *IR* value typically leads to poorer classification performance, especially for minority classes, and it is expressed as,

$$IR=\frac{N_{\min}}{N_{\max}}$$ (1)

where $N_{\max}$ and $N_{\min}$ denote the maximum and the minimum sample size in a set, respectively. The closer the *IR* value is to 1, the more balanced the dataset becomes. The specific *IR* ratios of Lychee13-3634 are shown in Table 3. It can be found that the *IR* value of ’single’ category (image only contains a single lychee) and ’multiple’ category (image contains multiple lychees of the same variety) is 3.6 (the maximum category is ’single’, the minimum category is ’multiple’). The *IR* of the entire dataset (both ’single’ and ’multiple’ categories) is 1.6 (the maximum category is ’HangingGreen lychee’, the minimum category is ’Guiwei lychee’). The *IR* of only the ’single’ category is 1.7 (the maximum category is ’HangingGreen lychee’, the minimum category is ’Guiwei lychee’), and the *IR* of only the ’multiple’ category is 1.9 (the maximum category is ’TopScorerRed lychee’ and the minimum category is ’Whitewax lychee’). In summary, Lychee13-3634 is a relatively balanced dataset compared to other fruit datasets. Data balance is crucial for ensuring that models perform well across all categories, especially for datasets with fewer categories.

**Table 3:** The *IR* analysis of Lychee13-3634.

Indicators	Category *IR*	Hybrid category *IR*	Single category *IR*	Multiple category *IR*
*N*_*mj*_	Single (2848)	HangingGreen_lychee (384)	HangingGreen_lychee (303)	TopScorerRed_lychee (84)
*N*_*min*_	Multiple (786)	Guiwei_lychee (233)	Guiwei_lychee (176)	Whitewax_lychee (44)
*IR*	3.6	1.6	1.7	1.9

## Experimental results on Lychee13-3634

To create a high-quality and highly representative dataset system for experimental validation, we adopt stratified random sampling to divide Lychee13-3634 into a fixed 80% (2907 images for training) and 20% (727 images for testing) to maintain the variety distribution similar to the original dataset.

### Image classification models

To validate the applicability and effectiveness of Lychee13-3634, we selected 16 representative or recent deep learning-based classification models [21–23], including ResNet152, ResNet101, ResNet50, ResNet34, ResNet18 [24], EfficientNetv2-s, EfficientNetv2-m, EfficientNetv2-l [25], SENet [26], Vision Transformer [27], Res-Att-Net [28], MobileViT [29], SqueezeNet [30], ShuffleNetv2 [31], MobileNetV2 [32], and MobileNetV4 [33], as well as 5 YOLO (-v5 [34], -v7 [35], -v8 [36], -v9 [37], and -v10 [38]) algorithms for experiments. These classifiers were chosen for their superior performance, lower computational complexity, and good generalization ability, particularly in classification tasks. Specifically, ResNet series solves the problem of gradient disappearance in traditional neural networks through residual connections and can conduct deeper training on lychee images and achieve better feature extraction. Moreover, different depth versions of ResNet can be flexibly adapted to the size of the dataset and computing resources, an advantage over many other architectures. Deep learning-based models have the unique advantages of attention mechanisms and lightweight design, meeting the different analysis requirements of the lychee dataset. YOLO series has excellent real-time object detection performance and can provide fast and accurate lychee classification results, which is difficult for most other architectures to achieve. All the compared methods were coded with the PyTorch framework. To ensure optimal performance of each model, we retained their original hyper-parameter settings, as detailed in Table 4. Notably, in the last column, the data marked as ’Fixed (8:2)’ means that the method uses a fixed split of 8:2 for training/testing sets.

**Table 4:** The detailed hyper-parameter settings of different models.

Hyper-parameter setttings	Models	tasks	epochs	bs	optimizer	lr	training/testing set
*Residual Network Models*	ResNet152	hybrid	30	16	Adam	1e-4	Fixed (8:2)
	ResNet101	hybrid	30	16	Adam	2e-4	Fixed (8:2)
	ResNet50	hybrid	150	16	Adam	1e-3	Fixed (8:2)
	ResNet34	hybrid	300	16	Adam	1e-1	Fixed (8:2)
	ResNet18	hybrid	250	128	SGD	1e-1	Fixed (8:2)
	Res-Att-Net	hybrid	184	64	SGD	1e-1	Fixed (8:2)
*Deep Learning Models*	EfficientNetv2-s	fruits	30	16	SGD	1e-2	random.seed(0)
	EfficientNetv2-m	fruits	30	16	SGD	1e-2	random.seed(0)
	EfficientNetv2-l	fruits	30	16	SGD	1e-2	random.seed(0)
	SENet	plants	300	4	Adam	1e-4	Fixed (8:2)
	VisonTransformer	plants	30	16	SGD	1e-3	random.seed(0)
	MobileViT	plants	30	8	Adam	1e-3	Fixed (8:2)
	SqueezeNet	hybrid	100	64	SGD	1e-3	random.seed(1)
	ShuffleNetv2	hybrid	200	32	Adam	1e-4	Fixed (8:2)
	MobileNetV2	hybrid	30	16	Adam	1e-3	Fixed (8:2)
	MobileNetV4	hybrid	100	128	AdamW	1e-3	Fixed (8:2)
*YOLO Series Models*	YOLOv5	/	100	16	SGD	1e-2	random.seed(100)
	YOLOv7	/	100	16	SGD	1e-2	random.seed(100)
	YOLOv8	/	100	16	SGD	1e-2	random.seed(100)
	YOLOv9	/	100	16	SGD	1e-2	random.seed(100)
	YOLOv10	/	100	4	AdamW	2e-3	random.seed(100)

### Experimental evaluation indicators

*Classification effectiveness evaluation indicators*. In this paper, we use common evaluation indicators [39,40], including Precision (Pre.), Recall [41] (Rec.), F1-Score [42] (F1.), Accuracy [43] (Acc.), top-1 and top-5 [44], to comprehensively evaluate and analyze the classification performance of different models on both the Fruits-360 and our Lychee13-3634. In particular, top-1 accuracy is the proportion of samples for which the model’s highest predicted probability class matches the actual class, while top-5 accuracy is that for which the actual class is among the top five predicted probability classes. The two indicators are particularly suitable for multi-classification tasks. They reflect the model’s performance in single predictions and candidate predictions, respectively. All the above evaluation indicators can respectively be defined as,

$$Precision=\frac{\left|TP\right|}{\left|TP\right|+\left|FP\right|}$$ (2)

$$Recall=\frac{\left|TP\right|}{\left|TP\right|+\left|FN\right|}$$ (3)

$$F1-score=2\times \frac{Precision\times Recall}{Presion+Recall}$$ (4)

$$Accuracy=\frac{\left|TP\right|+\left|TN\right|}{\left|TP\right|+\left|TN\right|+\left|FP\right|+\left|FN\right|}$$ (5)

*Model performance evaluation indicators*. The number of model parameters (Params) and the model running time (Times) are used for the model performance evaluation, where Params refers to the total number of trainable parameters contained in the model. It directly affects the model’s complexity and computational cost. In the lychee classification task, choosing a model with a moderate parameter count can reduce computational resource consumption while ensuring performance. And Times refers to the duration required for the model to make a prediction, including forward propagation and any necessary post-processing steps. In real-time application scenarios, time is a critical performance metric. Shorter prediction times mean the model can respond to queries more quickly, enhancing user experience.

### Lychee13-3634 evaluation

In this section, we apply 16 typical state-of-the-art deep learning-based models and 5 YOLO-series models for multi-class analysis on both our Lychee13-3634 and the existing Fruits-360.

#### Classification results on Lychee13-3634.

To comprehensively evaluate the effectiveness and reliability of the constructed Lychee13-3634, we introduced a variety of existing ResNet-based, deep learning-based, and YOLO-series models to carry out lychee image classification experiments. It is noted that for the YOLO series models, we first annotate the Lychee13-3634 and then use five common YOLO series models to carry out the lychee classification. The input image size is set to 256 × 256. The classification results are shown in Table 5, it can be found that SqueezeNet shows the worst performance with an accuracy of 71.53%. This may be due to its simple architecture, limited model capacity, and lack of targeted optimization for multi-classification tasks, resulting in unsatisfactory classification results for Lychee13-3634. In contrast, EfficientNetv2-s shows excellent performance with an accuracy of 99.99%. Although EfficientNetv2-m has the same performance as EfficientNetv2-s, it has significant expansion in parameter number (from 21.46M to 54.14M), which directly leads to the rise of its calculation cost and the extension of the average prediction time. For the YOLO series, it can be seen that all the YOLO algorithms achieve good classification performance on Lychee13-3634. In particular, YOLOv7 and YOLOv10 stand out in both precision and mAP50, with both values of 98%, showing a strong classification ability.

**Table 5:** The classification results of different models on Lychee13-3634.

Lychee13-3634	models	Acc.	Pre.	F1	Rec.	top1	top5
*ResNet Series*	ResNet152	98.89 ± 0.38	99.00 ± 0.72	98.43 ± 0.90	98.87 ± 0.77	98.87 ± 0.77	99.73 ± 0.27
	ResNet101	93.90 ± 0.87	96.30 ± 1.37	96.28 ± 1.38	95.66 ± 1.48	95.53 ± 1.50	99.73 ± 0.27
	ResNet50	89.10 ± 1.14	93.10 ± 1.84	93.61 ± 1.78	92.93 ± 1.86	92.81 ± 1.88	99.73 ± 0.27
	ResNet34	91.80 ± 1.00	94.00 ± 1.73	93.98 ± 1.73	93.97 ± 1.73	93.97 ± 1.73	99.73 ± 0.27
	ResNet18	85.95 ± 1.27	88.90 ± 2.28	88.38 ± 2.33	88.31 ± 2.34	88.95 ± 2.28	99.73 ± 0.27
	Res-Att-Net	99.64 ± 0.22	92.00 ± 1.97	91.00 ± 2.08	91.00 ± 2.08	91.64 ± 2.01	99.64 ± 0.44
*Deep*-*Learning Series*	EfficientNetv2-s	99.90 ± 0.09	99.73 ± 0.27	99.73 ± 0.27	99.73 ± 0.27	99.73 ± 0.27	99.73 ± 0.27
	EfficientNetv2-m	99.90 ± 0.09	99.73 ± 0.27	99.73 ± 0.27	99.73 ± 0.27	99.73 ± 0.27	99.73 ± 0.27
	EfficientNetv2-l	99.90 ± 0.09	99.00 ± 0.72	99.59 ± 0.40	98.18 ± 0.97	98.08 ± 1.00	99.73 ± 0.27
	SENet	98.63 ± 0.42	98.65 ± 0.84	98.62 ± 0.85	98.63 ± 0.85	98.63 ± 0.85	99.80 ± 0.19
	VisonTransformer	98.60 ± 0.43	98.96 ± 0.74	93.93 ± 1.74	98.91 ± 0.76	98.73 ± 0.81	99.73 ± 0.27
	MobileViT	96.84 ± 0.64	97.03 ± 1.23	96.62 ± 1.31	96.85 ± 1.27	96.85 ± 1.27	99.86 ± 0.13
	SqueezeNet	71.53 ± 1.64	70.86 ± 3.30	69.81 ± 3.34	70.00 ± 3.33	71.53 ± 3.28	98.04 ± 1.01
	ShuffleNetv2	80.10 ± 1.46	82.17 ± 2.78	83.06 ± 2.73	83.94 ± 2.67	84.32 ± 2.64	94.74 ± 1.62
	MobileNetV2	93.28 ± 0.91	93.54 ± 1.79	93.11 ± 1.84	93.16 ± 1.84	93.56 ± 1.78	99.73 ± 0.27
	MobileNetV4	89.86 ± 1.10	89.83 ± 2.20	89.45 ± 2.23	89.40 ± 2.24	89.86 ± 2.19	99.73 ± 0.27
*YOLO Series*	**models**	**Acc.**	**Pre.**	**F1**	**Rec.**	**mAP50**	**mAP50-95**
	YOLOv5	/	97.01 ± 1.24	97.23 ± 1.24	96.64 ± 1.42	99.07 ± 0.72	97.12 ± 1.24
	YOLOv7	/	98.51 ± 1.02	97.24 ± 1.24	97.33 ± 1.24	99.12 ± 0.72	97.59 ± 1.24
	YOLOv8	/	95.88 ± 1.58	94.88 ± 1.72	94.98 ± 1.72	91.91 ± 2.08	89.78 ± 2.27
	YOLOv9	/	88.82 ± 2.36	90.40 ± 2.18	93.09 ± 1.85	97.00 ± 1.24	95.09 ± 1.58
	YOLOv10	/	98.87 ± 1.02	98.60 ± 1.02	98.47 ± 1.02	99.02 ± 0.72	96.59 ± 1.42

**Fig 5:**
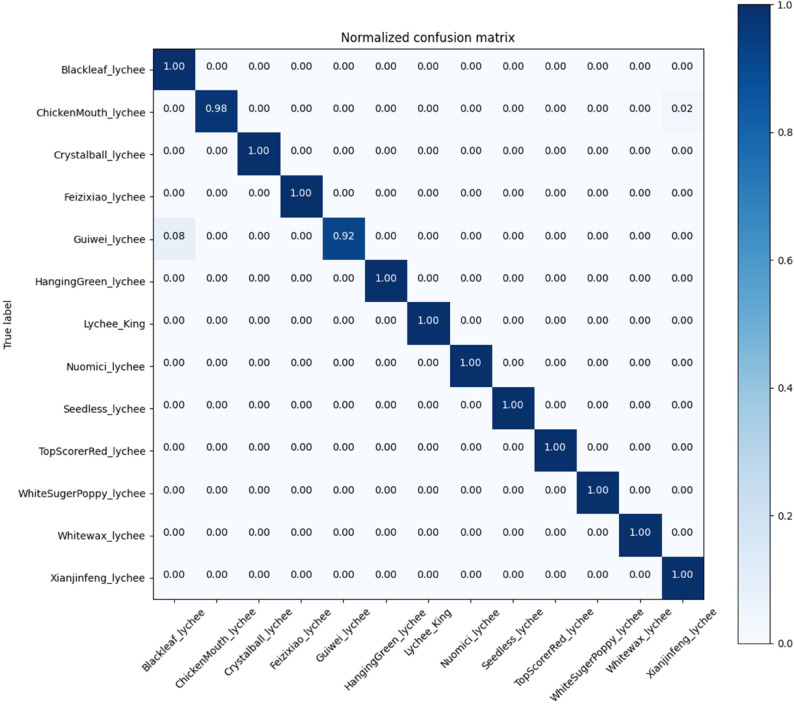
The confusion matrix of the best performing model EfficientNetv2-s. It can be found that except for the misclassification probability of 0.08 for ’ChickenMouth_1ychee’ as ’Xianjinfeng_lychee’ and 0.02 for ’Guiwei_1ychee’ as ’Blackleaf_lychee’, all other diagonal values are 1.0, which proves the strong strength and reliability of EfficientNetv2-s in lychee classification tasks.

**Fig 6:**
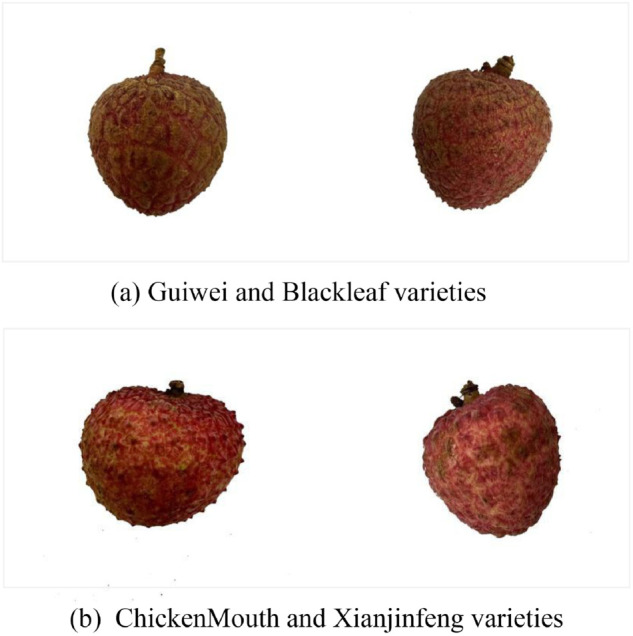
Two examples of common misclassify lychee varieties. (a) shows the easily confused Guiwei and Blackleaf varieties, which have similar textures and sizes. (b) shows the easily confused ChickenMouth and Xianjinfeng varieties, which have similar peel colors. This shows that the misclassification of lychees may not only be caused by the natural texture itself, but also by external factors such as freshness, angle, distance, and lighting when shooting.

To sum up, from the comprehensive consideration of classification accuracy and model efficiency, it can be considered that the EfficientNetv2-s model has more prominent advantages in the task of lychee image classification. To observe the excellent classification performance of EfficientNetv2-s more intuitively, we provide its confusion matrix as shown in Fig 5. It can be observed that the best model has the highest probability of misclassifying Guiwei as Heiye (8%), followed by misclassifying Chickenmouth as Xianjinfeng (2%). Fig 6 gives the visual examples of the common misclassified lychee varieties. It can be speculated that such misclassification is caused by the similarity of lychees themselves (Guiwei and Blackleaf), as well as uncontrollable factors such as light control, shooting distance, and freshness during the dataset acquisition process. This is also an important direction for the further improvement of the dataset in the future.

In addition, to verify whether the performance differences between different models are significant, we use K-fold cross validation (K=5 in this paper) for statistical testing, that is, through multiple iterations, each image can participate in both training and testing to avoid the random deviation of a single division. It is noted that, considering the uniformity of various indicators for different models and the fact that other indicators can be indirectly calculated through Acc. and Pre., we only use Acc. and Pre. as the result indicators of the statistical test of model differences. As shown in Table 6, the analysis of variance (ANOVA) based on the K-fold experimental results shows that the performance differences between the models are extremely statistically significant (F=189.42,p<0.001), that is, the average accuracy of high-performance models such as EfficientNetv2-l and SENet exceeds 99%, which is 2.46% higher than the average accuracy of medium-performance models such as ResNet-152 (96.56%), and the difference is significant, while the accuracy of lightweight models (such as SqueezeNet) is less than 75%, which is more than 15% lower than other models. Among the same type of models, there is no significant difference in performance between YOLOv5 and YOLOv9, but the former is more stable. VisionTransformer has a slight advantage over traditional CNN-based methods but is inferior to high-performance CNN-based methods. This indicates that there is a real gap in the model architecture’s ability to extract features from lychee images. In practical applications, models with significant advantages can be selected based on accuracy requirements and resource constraints.

**Table 6:** The average accuracy and precise of different models based on K-fold (K=5) cross validation.

Lychee13-3634	models	Aver_Acc.	Aver_Pre.
*ResNet Series*	ResNet152	96.96 ± 1.26	97.88 ± 1.06
	ResNet101	90.30 ± 2.17	91.10 ± 2.09
	ResNet50	89.83 ± 2.22	87.72 ± 2.23
	ResNet34	90.55 ± 2.14	90.90 ± 2.11
	ResNet18	85.01 ± 2.14	84.47 ± 2.26
	Res-Att-Net	86.70 ± 2.49	84.64 ± 2.64
*Deep*-*Learning Series*	EfficientNetv2-s	98.83 ± 0.79	98.31 ± 0.95
	EfficientNetv2-m	98.25 ± 0.96	97.97 ± 1.03
	EfficientNetv2-l	98.98 ± 0.74	98.67 ± 0.84
	SENet	99.09 ± 0.69	99.10 ± 0.69
	VisonTransformer	96.96 ± 1.26	98.26 ± 0.96
	MobileViT	97.94 ± 1.04	98.04 ± 1.02
	SqueezeNet	72.17 ± 3.28	71.57 ± 3.31
	ShuffleNetv2	80.75 ± 2.89	82.31 ± 2.80
	MobileNetV2	93.79 ± 1.77	93.72 ± 1.78
	MobileNetV4	86.47 ± 2.51	85.58 ± 2.57
*YOLO Series*	YOLOv5	98.52 ± 0.89	98.02 ± 1.02
	YOLOv7	81.82 ± 2.83	91.78 ± 2.01
	YOLOv8	94.40 ± 1.69	94.22 ± 1.71
	YOLOv9	96.08 ± 1.42	96.78 ± 1.29
	YOLOv10	98.60 ± 0.86	98.08 ± 1.01

#### Recognition results on Lychee13-3634.

YOLO-series models are renowned for their superior object recognition capabilities. To further validate the wide availability of Lychee13-3634, we perform the lychee image recognition task by training the five different YOLO models on it. The recognition results are shown in Table 7. It can be found that YOLOv10 gets the best comprehensive recognition results. To some extent, the data from Table 7 also reflects the applicability of Lychee13-3634 in image recognition.

**Table 7:** The recognition results of YOLO-series models on Lychee13-3634.

Lychee13-3634	models	Pre.	Rec.	mAP50	mAP50-95	true positive rate
*YOLO Series*	YOLOv5	0.999	1	0.995	0.801	0.903
	YOLOv7	1	0.988	0.993	0.780	0.762
	YOLOv8	0.998	0.988	0.990	0.791	0.639
	YOLOv9	0.999	0.988	0.992	0.806	0.593
	YOLOv10	1	0.999	0.995	0.820	0.965

#### Classification results on Fruits-360.

To verify the effectiveness of our Lychee13-3634 and explore the generalization ability of each model on different datasets, we used another dataset (Fruits-360 [12]), which is widely recognized in the field of fruit classification, to carry out an extended experiment. The Fruits-360 has a wide range of fruit types and rich images, which can provide a variety of test scenarios for the classification model.

The classification results on Fruits-360 are shown in Table 8, it is worth noting that EfficientNetv2-s once again shows excellent performance, and its accuracy is as high as 99.9%. It can be seen that in the field of classification of a variety of fruits or lychee, EfficientNetv2 shows the best effect and has the highest applicability. However, the performance of ResNet34 is relatively inferior, and its accuracy rate is 91.2%. Despite SqueezeNet performing poorly in the lychee classification, it shows adaptability to a variety of fruit classifications, which may be related to the differences in dataset characteristics. In summary, the classification results of each model on Lychee13-3634 are consistent with those on Fruits-360, which reflect the similarities and differences between the two datasets. This consistency strongly proves the effectiveness of the lychee dataset proposed in this paper in capturing lychee characteristics, and its generalization is equivalent to other widely recognized fruit datasets (such as Fruits-360). Notably, due to the huge amount of data in the Fruits-360 and the lack of label files directly applicable to the YOLO-series model, we did not use the YOLO-series model for testing in this study.

**Table 8:** The classification results of different models on Fruits-360.

Fruits-360	Model	Acc.	Pre.	F1	Rec.	top1	top5
*ResNet Series*	ResNet152	99.80 ± 0.034	99.80 ± 0.058	99.83 ± 0.054	99.86 ± 0.049	99.89 ± 0.043	99.96 ± 0.026
	ResNet101	98.92 ± 0.078	98.80 ± 0.141	98.72 ± 0.146	98.64 ± 0.151	98.76 ± 0.144	99.87 ± 0.049
	ResNet50	93.98 ± 0.18	95.89 ± 0.258	95.46 ± 0.271	95.04 ± 0.283	94.68 ± 0.292	99.73 ± 0.068
	ResNet34	91.20 ± 0.21	94.00 ± 0.310	93.28 ± 0.326	92.58 ± 0.341	92.50 ± 0.342	99.45 ± 0.096
	ResNet18	98.92 ± 0.078	99.01 ± 0.129	98.93 ± 0.134	98.90 ± 0.136	98.92 ± 0.134	99.99 ± 0.01
	Res-Att-Net	96.52 ± 0.14	74.00 ± 0.572	62.00 ± 0.631	66.00 ± 0.616	65.73 ± 0.618	96.52 ± 0.238
*Deep*-*Learning Series*	EfficientNetv2-s	99.90 ± 0.024	99.90 ± 0.041	99.90 ± 0.041	99.90 ± 0.041	99.99 ± 0.01	99.99 ± 0.01
	EfficientNetv2-m	99.90 ± 0.024	99.90 ± 0.041	99.90 ± 0.041	99.90 ± 0.041	99.99 ± 0.01	99.99 ± 0.01
	EfficientNetv2-l	99.90 ± 0.024	99.90 ± 0.041	99.90 ± 0.041	99.90 ± 0.041	99.99 ± 0.01	99.99 ± 0.01
	SENet	98.30 ± 0.098	98.81 ± 0.141	98.27 ± 0.170	98.31 ± 0.168	98.31 ± 0.165	99.90 ± 0.041
	VisonTransformer	99.90 ± 0.024	99.89 ± 0.043	88.20 ± 0.421	78.96 ± 0.530	77.47 ± 0.544	96.26 ± 0.247
	MobileViT	99.86 ± 0.028	99.86 ± 0.049	99.84 ± 0.052	99.85 ± 0.052	99.85 ± 0.052	99.96 ± 0.026
	SqueezeNet	99.89 ± 0.025	99.89 ± 0.043	99.89 ± 0.043	99.89 ± 0.043	99.89 ± 0.043	99.99 ± 0.01
	ShuffleNetv2	99.16 ± 0.069	99.47 ± 0.095	99.60 ± 0.082	99.74 ± 0.066	99.52 ± 0.09	99.89 ± 0.043
	MobileNetV2	98.68 ± 0.086	98.65 ± 0.150	98.50 ± 0.158	98.60 ± 0.153	98.60 ± 0.153	99.82 ± 0.055
	MobileNetV4	98.73 ± 0.084	98.90 ± 0.136	98.63 ± 0.151	98.73 ± 0.146	98.73 ± 0.146	99.97 ± 0.022

Based on experimental results on Lychee13-3634 and Fruits-360, it can be observed the significant differences in performance between different models. Specifically, in terms of classification performance, Resnet-152 successfully alleviated the gradient disappearance problem due to its sufficient depth and effective residual structure. Res-Att-Net is more consistent with the data characteristics. This demonstrates that the feature distribution and complexity of the dataset have a direct impact on the model performance. EfficientNetv2 series performs well in both datasets, demonstrating the wide adaptability of its architecture to different datasets. VisionTransformer has a low recall on Fruits-360, probably because of its weak ability to capture local detailed features. In terms of parameter optimization, the general strategy is dynamically adjusting the learning rate, such as using a large learning rate at the initial stage of training to converge quickly, while reducing the learning rate at the later stage to avoid missing the optimal solution. The weight initialization can be selected according to model structure. For instance, residual network adjusts jump connections or adds batch normalization, and EfficientNetv2 series optimizes data enhancement strategy to further improve model performance.

### Model efficiency evaluation

In addition, we also comprehensively compare the parameter number and running time of each model on both Lychee13-3634 and Fruits-360, as shown in Table 9. On Lychee13-3634, the Res-Att-Net had the shortest average classification time (0.0011s), followed by MobileNet (0.0014s). Although EfficientNetv2-l has excellent classification performance, the average time of it is the longest (1.0764s). On Fruits-360, SqueezeNet has the shortest average time (0.0001s). Similarly, the EfficientNet series still takes the longest time (0.9758s). In terms of the parameter number, SqueezeNet is the least (0.73M). The EfficentNetv2-l model has the most parameters (118.52M). In summary, EfficientNetv2 has the best classification performance, but it also has the longest running time and the most parameters.

**Table 9:** Comparisons of parameter number and time consumption of different models on both Lychee13-3634 and Fruits-360.

Efficiency Comparisons	models	Params (M)	Times (s)	
			Lychee13-3634	Fruits-360
*Residual Network Models*	ResNet152	60.19	0.1647	0.1265
	ResNet101	44.55	0.1327	0.0821
	ResNet50	25.56	0.0936	0.0585
	ResNet34	21.80	0.0847	0.0022
	ResNet18	11.69	0.0016	0.0017
	Res-Att-Net	19.92	0.0011	0.0015
*Deep Learning Models*	EfficientNetv2-s	21.46	0.2344	0.1970
	EfficientNetv2-m	54.14	0.5932	0.4173
	EfficientNetv2-l	118.52	1.0764	0.9758
	SENet	4.21	0.0459	0.0618
	VisonTransformer	103.19	0.1377	0.1688
	MobileViT	1.27	0.0015	0.0525
	SqueezeNet	0.73	0.2400	0.0001
	ShuffleNetv2	2.28	0.0019	0.0113
	MobileNetV2	3.50	0.0014	0.0354
	MobileNetV4	2.54	0.0004	0.0045
*YOLO Series Models*	YOLOv5	20.90	0.0128	0.0150
	YOLOv7	36.54	0.0109	0.0147
	YOLOv8	25.84	0.0070	0.0049
	YOLOv9	50.72	0.0445	0.0618
	YOLOv10	2.69	0.0774	0.0606

### Ablation studies

In this section, we conducted ablation studies on different background preprocessing and dataset balance of Lychee13-3634.

#### Impact of dataset preprocessing on model.

To evaluate the impact of different background preprocessing techniques on the classification performance, we compared the accuracy of three different models (SqueezeNet, EfficientNetV2-S, and YOLOv8) without background preprocessing, black background preprocessing, and white background preprocessing, respectively. The three selected models cover classifiers of different performance levels, and thus it can more comprehensively analyze the impact of background processing and model architecture on classification performance.

As shown in Table 10, it can be found that background processing has a significant impact on model performance. Specifically, white background preprocessing can improve the classification accuracy, while black background preprocessing leads to performance degradation. Moreover, the adaptability of different models to background changes varies. EfficientNet shows the most stable performance under different background treatments. SqueezeNet is sensitive to background changes. YOLOv8 performs well in unprocessed backgrounds, but has limited adaptability in black backgrounds.

**Table 10:** The accuracy comparison of three typical models based on different background preprocessing for Lychee13-3634.

Models	background type	Acc.	Pre.	F1	Rec.	top1	top5
*SqueezeNet*	w/o preprocessing	78.62	78.55	77.80	78.27	78.62	98.57
	black background	66.16	67.26	65.07	65.72	66.16	94.34
	white background	71.53	70.86	69.81	70.00	71.53	98.04
*EfficientNet*	w/o preprocessing	99.72	100	100	100	99.72	100
	black background	98.4	90.51	91.27	91.73	90.51	99.30
	white background	99.90	100	100	100	100	100
*YOLOv*8	w/o preprocessing	100	100	100	100	100	100
	black background	84.60	79.90	82.00	85.10	84.60	90.90
	white background	94.10	93.90	93.70	93.80	94.10	100

#### Impact of dataset *IR* on model.

To evaluate the impact of dataset balance on model classification performance, we aligned the number of samples in all categories of Lychee13-3634 with the category with the smallest number of samples in Lychee13-3634, and constructed a balanced dataset. We respectively chose one algorithm from the ResNet series (ResNet50), deep learning series (MobileViT), and YOLO series (YOLOv8) as an example to conduct an ablation study.

As shown in Table 11, after dataset balancing, the accuracy, precision, and F1-score of the three models have been improved to varying degrees. This indicates that the balance of the dataset is crucial for improving the generalization and classification performance of the model. Future research will further explore how to optimize the balance of the dataset through data augmentation and sampling techniques, thereby improving the robustness and classification accuracy of the model.

**Table 11:** The accuracy comparison of 3 typical models under different *IR* of Lychee13-3634.

Models	*IR* value	Acc.	Pre.	F1	Rec.	top1	top5
*ResNet*50	1.6	89.10	93.10	93.61	92.93	92.81	100
	1	90.65	93.73	93.79	93.83	93.61	100
*MobileViT*	1.6	96.84	97.03	96.62	96.85	96.85	99.86
	1	97.72	98.00	97.90	97.90	97.90	100
*YOLOv*8	1.6	94.41	93.90	93.70	93.80	94.1	100
	1	94.42	94.40	94.20	94.20	94.20	100

## Conclusion

In this paper, we construct a comprehensive and diverse benchmarking lychee image dataset (Lychee13-3634), which consists of 13 varieties and 3634 images with a uniform format. To our knowledge, Lychee13-3634 is currently the only standard and publicly available dataset for lychee image classification. To demonstrate the availability and effectiveness of Lychee13-3634, we conducted classification experiments on 21 typical and recent classification models based on it and the existing fruit dataset (Fruits-360). Experimental results show that different models have similar classification performance on the two datasets, which reflects the usability of our Lychee13-3634. Besides, we also conduct ablation studies on the different preprocessing technologies and dataset *IR*, and provide meaningful insights for laying the foundation for future lychee classification algorithms. Although Lychee13-3634 shows good universality in classification tasks, it still has certain limitations, such as the lack of information on lychee maturity, size, and harvesting location, which may limit its applicability in certain research scenarios. Besides, since the experiments were conducted under controlled laboratory conditions, it may not fully represent the challenges encountered in real-world agricultural deployment scenarios. Therefore, future work will focus on investigating the model robustness under varying field conditions to bridge the gap between laboratory and real-world applications.
